# Ecosystem Model Skill Assessment. Yes We Can!

**DOI:** 10.1371/journal.pone.0146467

**Published:** 2016-01-05

**Authors:** Erik Olsen, Gavin Fay, Sarah Gaichas, Robert Gamble, Sean Lucey, Jason S. Link

**Affiliations:** 1 Institute of Marine Research, PB 1870 Nordnes, N-5817, Bergen, Norway; 2 NOAA Northeast Fisheries Science Center, 166 Water St., Woods Hole, Massachusetts, 02543–1026, United States of America; 3 School for Marine Science and Technology, University of Massachusetts Dartmouth, 200 Mill Road, Fairhaven, Massachusetts, 02719, United States of America; 4 NOAA, National Marine Fisheries Service, 166 Water Street, Woods Hole, Massachusetts, 02543, United States of America; Università di Genova, ITALY

## Abstract

**Need to Assess the Skill of Ecosystem Models:**

Accelerated changes to global ecosystems call for holistic and integrated analyses of past, present and future states under various pressures to adequately understand current and projected future system states. Ecosystem models can inform management of human activities in a complex and changing environment, but are these models reliable? Ensuring that models are reliable for addressing management questions requires evaluating their skill in representing real-world processes and dynamics. Skill has been evaluated for just a limited set of some biophysical models. A range of skill assessment methods have been reviewed but skill assessment of full marine ecosystem models has not yet been attempted.

**Northeast US Atlantis Marine Ecosystem Model:**

We assessed the skill of the Northeast U.S. (NEUS) Atlantis marine ecosystem model by comparing 10-year model forecasts with observed data. Model forecast performance was compared to that obtained from a 40-year hindcast. Multiple metrics (average absolute error, root mean squared error, modeling efficiency, and Spearman rank correlation), and a suite of time-series (species biomass, fisheries landings, and ecosystem indicators) were used to adequately measure model skill. Overall, the NEUS model performed above average and thus better than expected for the key species that had been the focus of the model tuning. Model forecast skill was comparable to the hindcast skill, showing that model performance does not degenerate in a 10-year forecast mode, an important characteristic for an end-to-end ecosystem model to be useful for strategic management purposes.

**Skill Assessment Is Both Possible and Advisable:**

We identify best-practice approaches for end-to-end ecosystem model skill assessment that would improve both operational use of other ecosystem models and future model development. We show that it is possible to not only assess the skill of a complicated marine ecosystem model, but that it is necessary do so to instill confidence in model results and encourage their use for strategic management. Our methods are applicable to any type of predictive model, and should be considered for use in fields outside ecology (e.g. economics, climate change, and risk assessment).

## Introduction

Analyzing the effects of multiple pressures on complex socio-ecological systems is challenging, requiring advanced quantitative tools to capture interactions and feedbacks. Mathematical models of ecosystems offer one means to address this complexity and allow for simulation of a range of possible future system states under alternative management actions. ***Ecosystem models have the potential to inform management of the impacts of human activities in a changing environment*, *but can we rely on them***? The key question that needs answering is: how good is ecosystem model skill at forecasting future conditions?

Many types of mathematical models are applied in forecasting. These include physical models of the atmosphere for weather forecasts [1], hydrodynamic models for marine and coastal forecasts [2], and population dynamics models of commercially exploited fish populations for fisheries management [3]. Skill assessment, also termed validation, determines how well a model reproduces the true system state[2,4]. Here we distinguish between forecast skill and hindcast skill. Hindcast skill can be evaluated by comparing model outputs to historical data, often used to statistically estimate values for model parameters [5–7]. However, a model that has high hindcast skill will not necessarily have high forecast skill when confronted with data outside of that used for parameter estimation [8]. Hindcast skill assessment is useful for building and tuning a model, but the ability of models to reliably forecast future conditions is essential for use in decision-making. Determining model forecast skill involves building and tuning a model, using it to forecast future conditions, and then, in the future, evaluating how the model predictions compare with the (now available) observed data. Models used for weather forecasting are routinely subject to skill assessment, as observational data to test forecast skill is available within hours or days of the forecast being made. Evaluating forecast skill is a rigorous test of model performance typically used in meteorological model skill assessments [9], and the earth-system models [10][1,11]. For systems requiring prediction over longer timescales (e.g. years) there are often fewer observations with which to evaluate skill, and the lag between model prediction and observation becomes much longer. Skill assessment methods also assume that the true state of the system lies within observational uncertainty. Process error and structural uncertainty add to this system uncertainty. Skill assessment is made more difficult if observational data are missing, or future states of the system lie outside the range of previously observed values. These factors may account for the rarity of skill assessment applied to complex ecosystem models.

In fisheries and ocean management, end-to-end ecosystem models that attempt to integrate all relevant aspects of ocean physics and chemistry, primary productivity, nutrient cycling, zooplankton, commercial and un-harvested fish and invertebrates, marine reptiles, mammals, seabirds, and human pressures on the system are increasing in development [12–16]. The goal of end-to-end models to be comprehensive in covering the entire system distinguishes them from simpler ecosystem models that typically only include selected parts and exclude many components and processes [17]. These end-to-end models are mainly designed to develop scenarios for ecosystem-level Management Strategy Evaluation [16,18] to guide holistic decision-making on balancing harvest of different species or different fleet structures to name a few. This is in contrast to providing tactical management advice for individual species or habitats. Ecosystem models need to adequately characterize the ecological relationships between system components (individual species or functional groups) and how these components co-vary under different ecosystem drivers and pressures (e.g. harvesting, climate, and management interventions). Further, we need to know how well the model predicts emergent ecosystem properties such as total system productivity and the mean trophic level of the community, which are frequently proposed as indicators of ecosystem status for ecosystem based management [19–22].

**Fig 1:**
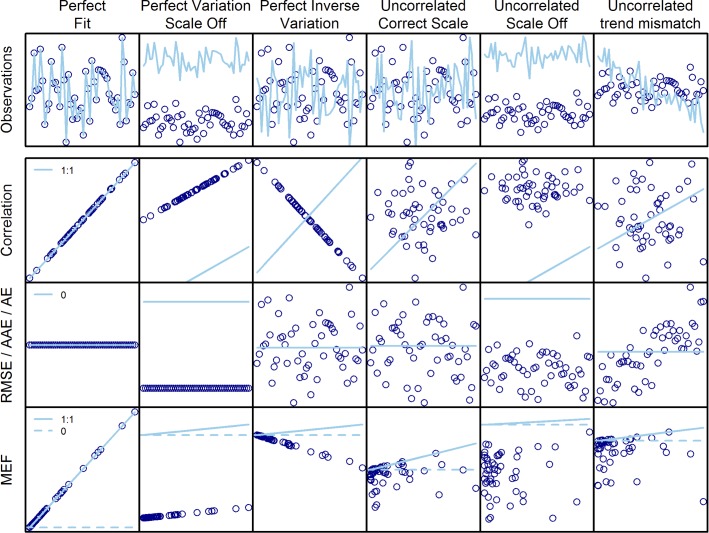
Conceptual Fig comparing the skill metrics (RMSE, AAE, AE, MEF and correlation) performance using simulated observed (open circles) and modeled data (lines).

Skill assessment of an end-to-end ecosystem model requires a comparison of model output to observations across the modeled species and ecological processes (emergent properties). Multiple metrics are often applied to assess model skill because these metrics may emphasize different aspects of skill. Calculation of skill assessment metrics include both univariate and multivariate methods, with most applications comparing model outputs with observational data at different spatial and temporal scales [23–25]. Understanding which aspects of model performance the different metrics address can be difficult. To deal with this, we developed a conceptual model to compare the metrics we use in the skill assessment of an end-to-end marine ecosystem model (Fig 1).

This paper demonstrates the first comprehensive forecast skill assessment for an end-to-end ecosystem model, the Northeast U.S. (NEUS) Atlantis model that was developed by Link et al. [19], illustrating the practical use of the skill assessment methods presented above. The NEUS model was tuned to survey data and stock assessment output from 1964–2004. We address skill assessment by comparing the NEUS model 40-year hindcasts (pre 2004) and 10-year forecasts (2004–2013) with survey data collected from 1964 through 2013, as these data are the best available proxy for the true system status. The NEUS continental shelf marine ecosystem has rich observational data for multiple species, derived from semiannual trawl surveys of the entire shelf as well as other monitoring programs.

We move beyond the more typical assessment of single component skill (e.g. asking whether biomass trends for individual species are predicted correctly) by evaluating model skill at forecasting emergent ecosystem properties. We use time series of data-derived ecosystem indicators to represent these emergent ecosystem properties. Indicators selected have been assessed and vetted [20,21,26,27] as illuminating and capturing key system properties. We then evaluate the model’s ability to forecast indicators of emergent ecosystem properties in the same way as we evaluate its ability to forecast single components, using multiple metrics of model skill. We discuss the possibility of applying these methods in other settings and to other models, with particular relevance for strategic issues like integrated ecosystem assessment and management.

## Materials and Methods

**Fig 2:**
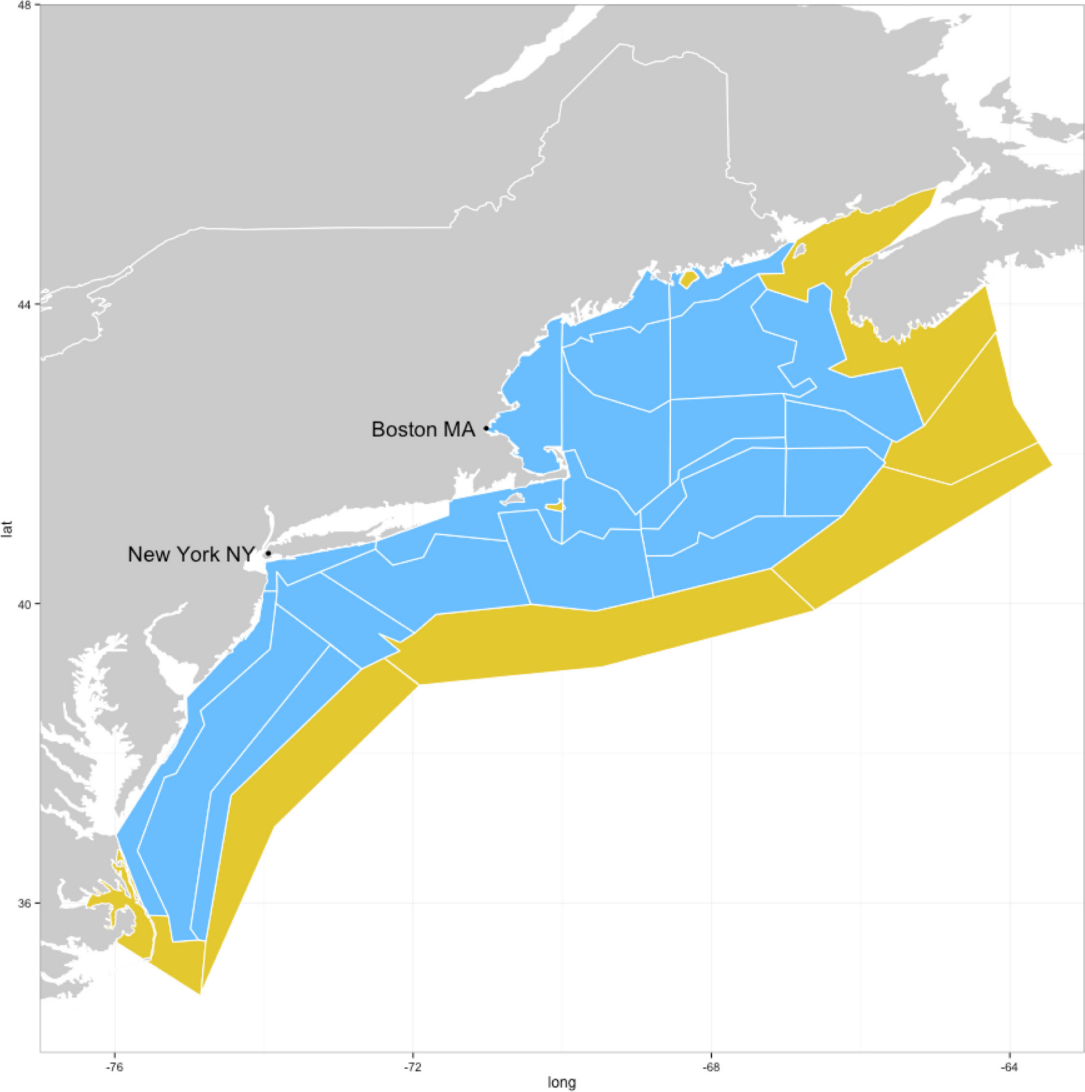
Map showing the Northeast (NE) USA and the Atlantis ecosystem model regions (blue) and boundary boxes (yellow).

The Atlantis model is a spatially explicit model, covering facets ranging from energy input to harvest (fisheries) and management [12,14–16] as well as being capable of handling other anthropogenic and climatic pressures [28,29]. The NEUS Atlantis model (Fig 2) [19] was the first full system Atlantis model developed outside of Australia, and the first to be calibrated to extensive data sets. The NEUS Large Marine Ecosystem (LME) covers an area of 293,000 km^2^ from the Gulf of Maine to Cape Hatteras and is comprised of four major sub-regions, each with their own distinct geophysical, biological, and human processes: Gulf of Maine, Georges Bank, Southern New England, and Mid-Atlantic Bight. The NEUS LME is highly productive, although production tends to be higher nearshore and in the northern sub-regions, and it has supported significant commercial fisheries for centuries[30]. The NEUS LME has seen a large shift from a community dominated by groundfish to one dominated by pelagic fish and elasmobranchs over the past 50 years [30]. The NEUS LME has long time-series of survey and fisheries monitoring data that are very useful for setting up and evaluating the skill of ecosystem models.

### Northeast U.S. (NEUS) model description

The NEUS Atlantis model area follows the LME area and is resolved into 22 spatial regions, each of which is further resolved by depth (Fig 2) into 5 water-mass layers as well as the sea-surface and sea-floor (epibenthic) where model processes occur. Eight boundary boxes (including islands) representing areas outside the model domain are also included. These boundary boxes do not have the full range of processes that are simulated in the other dynamic boxes, except for advection and diffusion from the dynamic boxes to the boundary boxes. The boundary boxes also incorporate processes for some groups related to seasonal migration to and from the model domain, and for growth and reproduction that occur outside of the model domain. The transport model, used to simulate advective and diffusive transport of nutrients and particles across the regions, as well as temperature and salinity within the water column in each region, was parameterized using the HYCOM model (www.hycom.org). We simulated the dynamics of 45 functional groups comprised of 18 fish groups, 6 groups of marine mammals, turtles, and birds, 19 invertebrate groups, and 6 detritus groups. Shrimp and squid were parameterized with separate adult and juvenile stages while fishes were parameterized with 10 stages (each 10% of the average life span for the group). Nitrogen is the common currency in an Atlantis model, but standard outputs of a model run include back-converted biomasses. An exploitation sub-model is comprised of eighteen fishing fleets that are combinations of fishing gears and target species. Each fleet was assigned target, bycatch, and discard groups among the vertebrate and invertebrate functional groups.

The model was calibrated to data from the Northeast U.S. from 1963–2004, primarily using information from the semiannual Northeast Fisheries Science Center (NEFSC) bottom trawl survey [31] and the NEFSC commercial fisheries database (NEFSC unpublished data) combining data across seasons. Four levels of calibration were done to achieve a base scenario (see [19,32] for details). The first level was biophysical with the goal of not allowing any functional group to go extinct. The second level was forcing catch with the observed time series of each fleet, and varying the biological parameters to tune the functional group biomasses to generally match surveys and stock assessment outputs. The third level was forcing fishing effort with the observed time series of each fleet, and varying the catch parameters to tune the catch to observed data. The final level was varying the fishing effort parameters to tune the effort to observed data for the whole model area. Particular attention and effort during calibration to achieve correspondence between model biomass and fishery output with observed survey and catch data was given to the following fish species of economic and ecological importance: silver hake, yellowtail flounder, cod, haddock, herring, spiny dogfish, and white hake. At each level of calibration the modelers attempted to, as much as possible, leave previously calibrated parameters alone. This was not always possible as new interactions were sometimes revealed, necessitating a revisiting of parameters that were calibrated at a previous level. The final base case scenario was run for 50 years (from 1964 to 2014). Comparative data used when calibrating the model was available from 1964 to 2004.

### Data

For forecast skill evaluation biomass and landings data were updated to include the time period from 2004–2013 using the same methods that were used during the initial calibration of the model (see Table 1). Biomass estimates were derived from the NEFSC bottom trawl survey [31]. In 2009, the NEFSC transitioned from the NOAA Ship Albatross IV to the NOAA Ship Henry B. Bigelow. Appropriate conversion factors were used so that the data collected using the new vessel was comparable to that collected with the old vessel [33]. Stratified mean catch rates were expanded to minimum swept-area biomass estimates by assuming a catchability of 1. We calculated typical 95% confidence bands around the mean survey biomass (mean +- 1.96 * std. dev.). In this case the mean is a minimum swept area estimate of the survey's stratified mean weight per tow. The variance calculation followed [34] for stratified sampling and was expanded to the minimum swept area estimate by multiplying the variance by the squared constant (qA/a) where q is the catchability coefficient, A is the total area, and a the swept area. Landings data were obtained from the commercial fisheries database maintained by the Greater Atlantic Regional Fisheries Office (GARFO). Seal population numbers and the whale population size data (right whales) were obtained from the upcoming *Ecosystem Status Report for the Northeast U*.*S*. *Continental Shelf Large Marine Ecosystem* (NOAA Northeast Fisheries Science Center, unpublished data). Average phytoplankton density as determined from satellite observations (SeaWIFS and MODIS, [35]) were used as data on primary production. Biomass and landings data were normalized for the direct comparison, but not when calculating the ecosystem indicators.

**Table 1:** Data uses, sources and years for which data are available that were collated and used as the observed data set in the model skill analysis.

Use	Data	Period
Fish biomass data	NOAA Northeast Fisheries Science Center bottom trawl survey biomass data	1964–2013
Fishery landings data	Greater Atlantic Regional Fisheries Office (GARFO)	1964–2013
Seal population numbers	*Ecosystem Status Report for the Northeast U*.*S*. *Continental Shelf Large Marine Ecosystem* (NOAA Northeast Fisheries Science Center, unpublished data)	1981–2013
Right whale population size	*Ecosystem Status Report for the Northeast U*.*S*. *Continental Shelf Large Marine Ecosystem* (NOAA Northeast Fisheries Science Center, unpublished data)	1990–2013
Average phytoplankton densities	SeaWIFS and MODIS satellite observations	1998–2013

### Ecological indicators

We calculated 22 ecosystem indicators that have previously been vetted and evaluated [20,22,27,36]. These were calculated from Atlantis model outputs (see Table 2) used in the present analysis. Mean trophic level values were derived from the NEFSC Food Web Dynamics Program food habits database [37,38]. Average catch value (U.S. $) per species for 2008 was used to calculate the value of the catch.

**Table 2:** List of ecosystem indicators calculated from the NEUS model data and the survey biomass and observed landings data.

Ecological indicators	
Total Biomass	Total biomass of fish, benthos, marine mammals, seabirds and cephalopods.
Total Catch	Total catch of commercial fish and benthos.
Catch/Biomass	Total catch as proportion of total biomass.
Fish Biomass	Total biomass of fish species.
Demersal/Pelagic Ratio	Biomass of all demersal fish as a proportion of biomass of all pelagic fish.
TEPs	Threatened, endangered, and protected species
Seal Biomass	Total biomass of seals
Whale Biomass	Total biomass of whales
Demersal Catch	Demersal fish catch (weight)
Pelagic Catch	Pelagic fish catch (weight)
MTL System	Mean trophic level of all the species in the system
MTL Catch	Mean trophic level of species in the catch
Biomass/PP	Total biomass as proportion of primary production
Demersal Biomass/PP	Demersal fish biomass as proportion of primary production
Pelagic Biomass/PP	Pelagic fish biomass as proportion of primary production
Catch/PP	Fisheries catch as proportion of primary production
Demersal Catch/PP	Demersal fish catch as proportion of primary production
Pelagic Catch/PP	Pelagic fish catch as proportion of primary production
Fish Catch	Catch of commercial fish (no scallops or lobster)
Fish Catch/Fish Biomass	Catch of commercial fish as proportion of total fish biomass
Proportion Overfished	Proportion of commercial species with low relative biomass at the end of the time-series
Value	Total value of the catch

### Skill metrics

Skill assessment involves comparing model outputs with observational data. Stow et al. (2009) [25] discussed 6 different metrics for model skill performance: average error (AE), average absolute error (AAE), root mean squared error (RMSE), modeling efficiency (MEF), correlation coefficient (S,P or K), and reliability index (RI); and recommended that several metrics be used “in concert, to provide a more thorough appraisal”. In a slightly different application, Fulton et al. (2005) [21] used only one of these metrics, correlation (both Pearson and Spearman), when evaluating indicator performance using ecosystem model output. Here we calculate five of the six metrics discussed by Stow et al. (2009) [25] and three variants of correlation: Spearman, Pearson, and Kendall (see Table 3) to allow for comparison among them. The reliability index was not calculated because it takes the log of observations divided by predictions. Since we standardized the observations and predictions, this would require taking the log of negative numbers.

**Table 3:** Skill metrics used in the analysis of ecosystem model skill.

Skill Metric	
AE	Average Error
AAE	Average Absolute Error
RMSE	Root Mean Squared Error
MEF	Modeling Efficiency
S	Spearman Rank Correlation
P	Pearson Correlation
K	Kendall Rank Correlation

The seven selected skill metrics would be expected to show a varied response to different types of mismatch between observed and modeled data, illustrated in the conceptual Fig 1. Across the top row of Fig 1 simulated time series of observations and model predictions compare perfect fit and multiple forms of poor fit including scale mismatch, inverse variation, lack of correlation, trend mismatch, and combinations thereof. Down the leftmost column we visualize perfect fit according to each class of metric (correlation, RMSE, AAE, AE, and MEF). In the second row from the top, observed data (x axis) are plotted against model predictions (y-axis) to evaluate correlation. The third row from the top shows time series plots of error (observed—model predicted) which form the basis for RMSE, AAE, and AE calculations. In the bottom row, we visualize modeling efficiency, which evaluates whether the model is an improvement over the mean of the observations. A perfect modeling efficiency aligns on the 1:1 axis, while the average of the observations aligns at y = 0; therefore, for the model to improve on the average of the observations (MEF>0), the points should be mainly between the horizontal line and the diagonal line on each plot. For correlations (row 2), AAE and AE (row 3) the straight line shows the ideal performance with no error.

Skill metrics were calculated for the three data-series for the following periods:

- Full model hindcast (1964–2004)
- No”spin up” (model stabilization) period hindcast (1974–2004) (results in Figs A–O in S1 File)
- Individual decadal scale hindcasts starting in 1964, 1974, 1984, and 1994 (results in Figs A–O in S1 File)
- Forecast period for which the model was not calibrated to data (2005–2014)

### Statistical analysis

All analyses and plotting were carried out in R-statistics [39] version 3.0.2 (“Frisbee Sailing”), run on Linux, OSX, and Windows machines. Data sets and R-code scripts for the analyses are freely available through GitHub: (https://github.com/erikjsolsen/AtlantisNEUS_R/tree/master/skill).

## Results

The conceptual model (Fig 1) shows that visual evaluation of observations relative to modeled time series can be difficult. For instance, it is very challenging to visually distinguish perfect inverse correlation of observations and model output from uncorrelated observations and output (Fig 1, top row, panels 3 and 4). However, the implications of each type of mismatch for model performance are very different. Additionally, each metric assesses a different aspect of skill, with scale mismatch (accuracy) being irrelevant to correlation but highly important for some metrics (RMSE, AAE, and MEF, Fig 1). However, some metrics are redundant as they essentially measure the same aspect of model skill performance (e.g. RMSE and AAE). Thus, providing many redundant measures of model skill will instill a false perception of the model’s performance (good or bad) leading to either unfounded trust or mistrust in the model results. Actual computed values of metrics for each of the simulated situations in our conceptual model also demonstrate tradeoffs between metrics. For example, there is a tendency for AE to indicate good fit because large negative and positive errors cancel each other [25,40].

**Fig 3:**
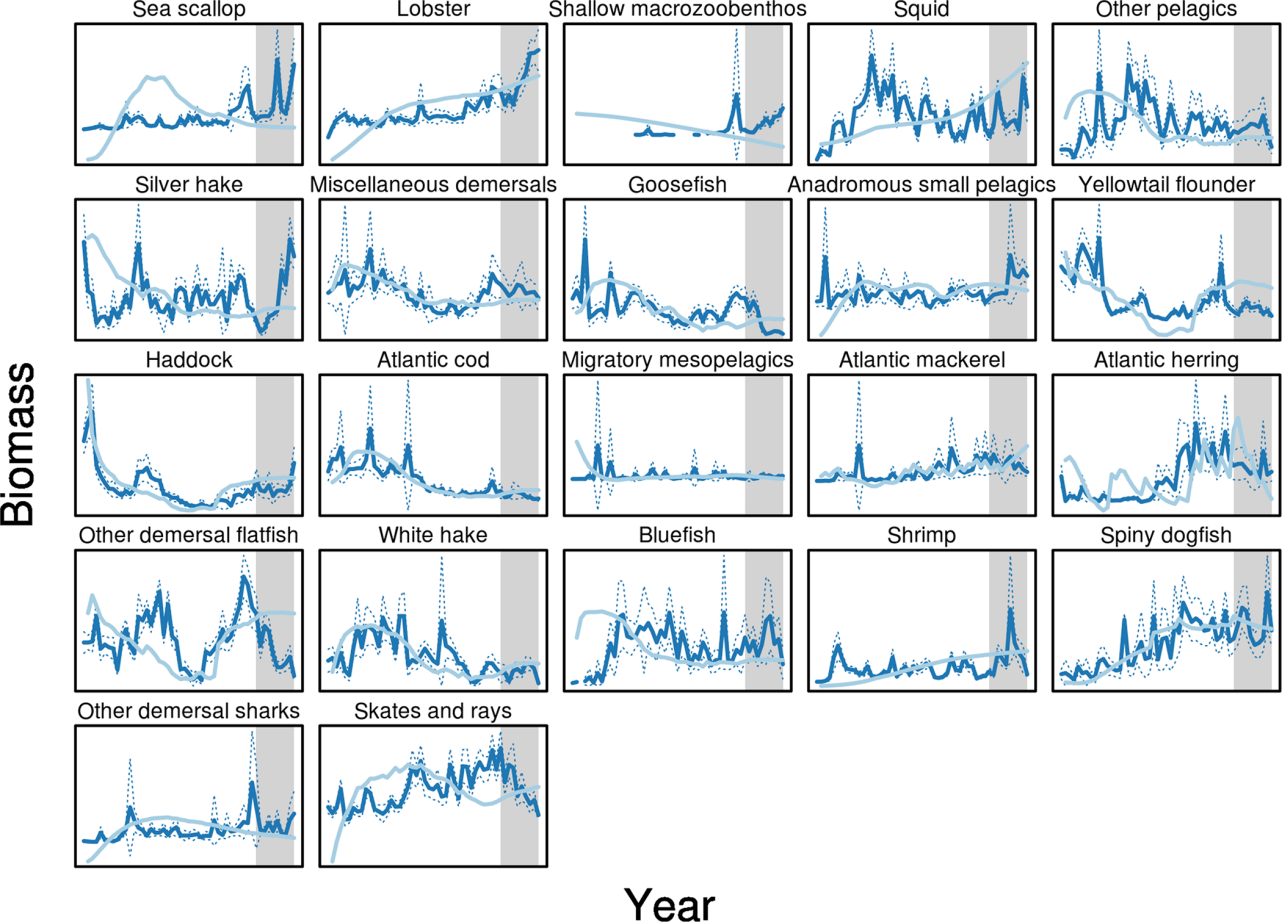
Biomass (normalized) time series. Survey data time series (dark blue) with 95% confidence interval bands (dotted lines) versus Atlantis model biomass predictions (light blue). Gray area indicates the forecast time-period from 2004–2013. We label (and color code) the data-categories Biomass (blue), Landings (orange), and Indicators (green), consistently throughout the results

**Fig 4:**
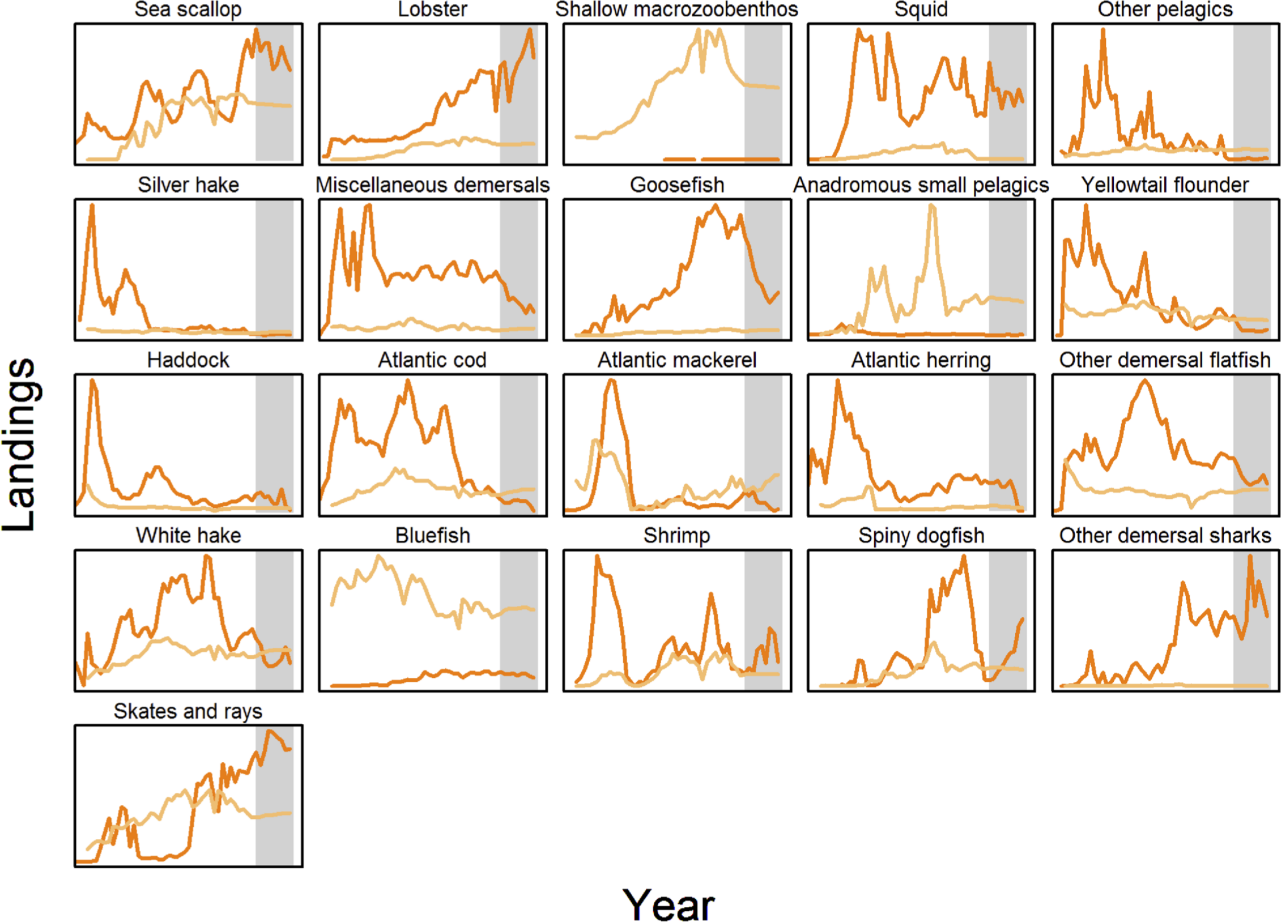
Landings (normalized) time series. Recorded (actual) landings (dark orange) versus Atlantis model catch predictions (light orange). Gray area indicates the forecast time-period from 2004–2013.

**Fig 5:**
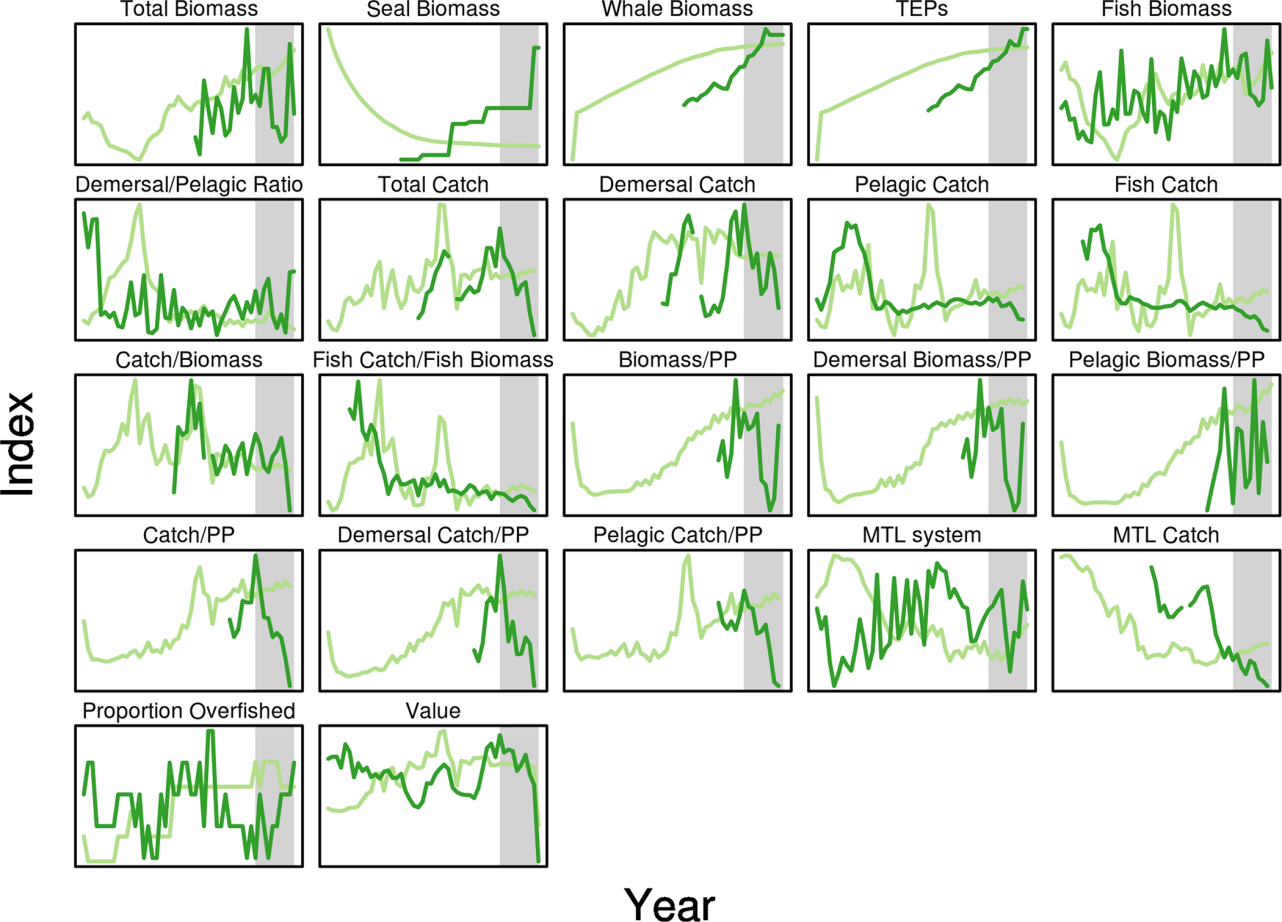
Time series of ecosystem indicators calculated using both Atlantis NEUS model data (dark green) and observed data (light green). Gray area indicates the forecast time-period from 2004–2013.

Visual inspection of time-series plots of model predictions versus observed data (Figs 3 and 4) shows that for some ecosystem components (e.g. biomass of cod, haddock, and spiny dogfish, Fig 3) the model and observed data correspond for both hindcast and forecast periods, mostly occurring within the observational uncertainty of the observed biomass time series (as shown by 95% confidence intervals). For other components there is little apparent correspondence between the model and observed data (e.g. biomass of squid and shrimp in Fig 3, landings of bluefish in Fig 4, or catch/biomass indicator, Fig 5). However, as noted for Fig 1, assessing model performance by subjective “eye-balling” is very difficult, as some parts of a time-series may be in better agreement between observed data and model predictions than others. We therefore emphasize results based on multiple metrics of model skill in both the hindcast and forecast periods. Objective model performance could only be evaluated using the MEF metric with its ‘0’ threshold that identifies when model results are better than a prediction of the average of the observed time-series. The other metrics had no such objective evaluation criteria and so were therefore of more use in relative skill evaluation between hindcast and forecast and amongst the modeled species and indicators.

### Quantifying model skill

Ecosystem models are often run for a number of years to "spin up" processes, stabilize interactions, remove undue influence of initial conditions on fast turnover properties (like phytoplankton biomass), and to allow for more reasonable age structures to become established. This could lead to differences in the model's ability to match observations at different points in the model time series. Neither visual inspection nor PCA analysis revealed any marked or time-dependent changes in model skill when calculated for the full hindcast period (1964–2003) or for each of the decades from 1964 separately (see Figs A–O in S1 File for details). We therefore compared the model forecast skill (2004–2013) to the model skill obtained for the full hindcast period (1964–2003) for biomass, landings, and indicators.

**Fig 6:**
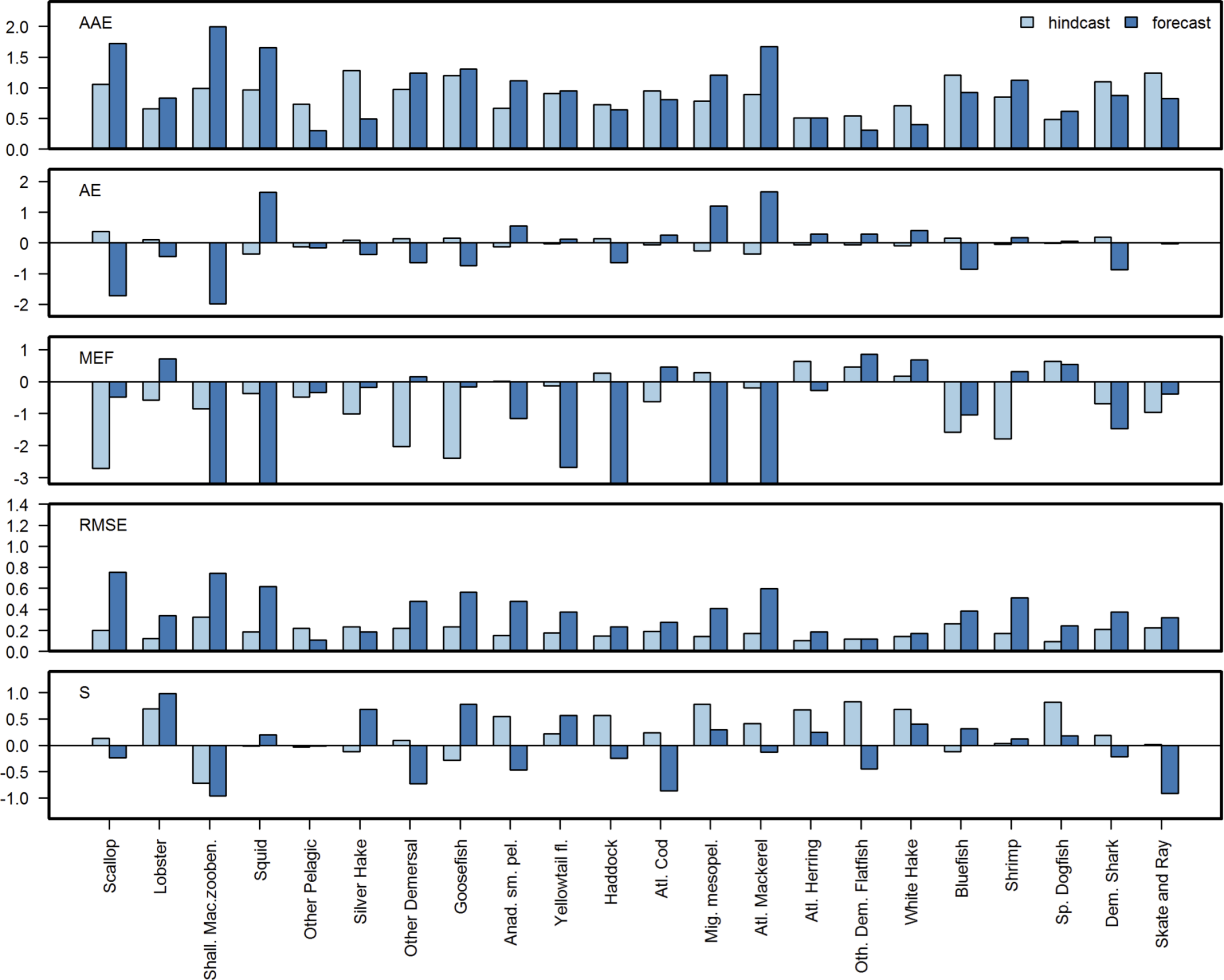
Forecast skill metrics for biomass data. Pairwise comparison of forecast and hindcast skill metric performance for 5 skill metrics: MEF, AE, AAE, RMSE and S(Spearman rank) Correlation for 22 species in the NEUS Atlantis ecosystem model. Y-axis limited to show values between -2 and 2.

**Fig 7:**
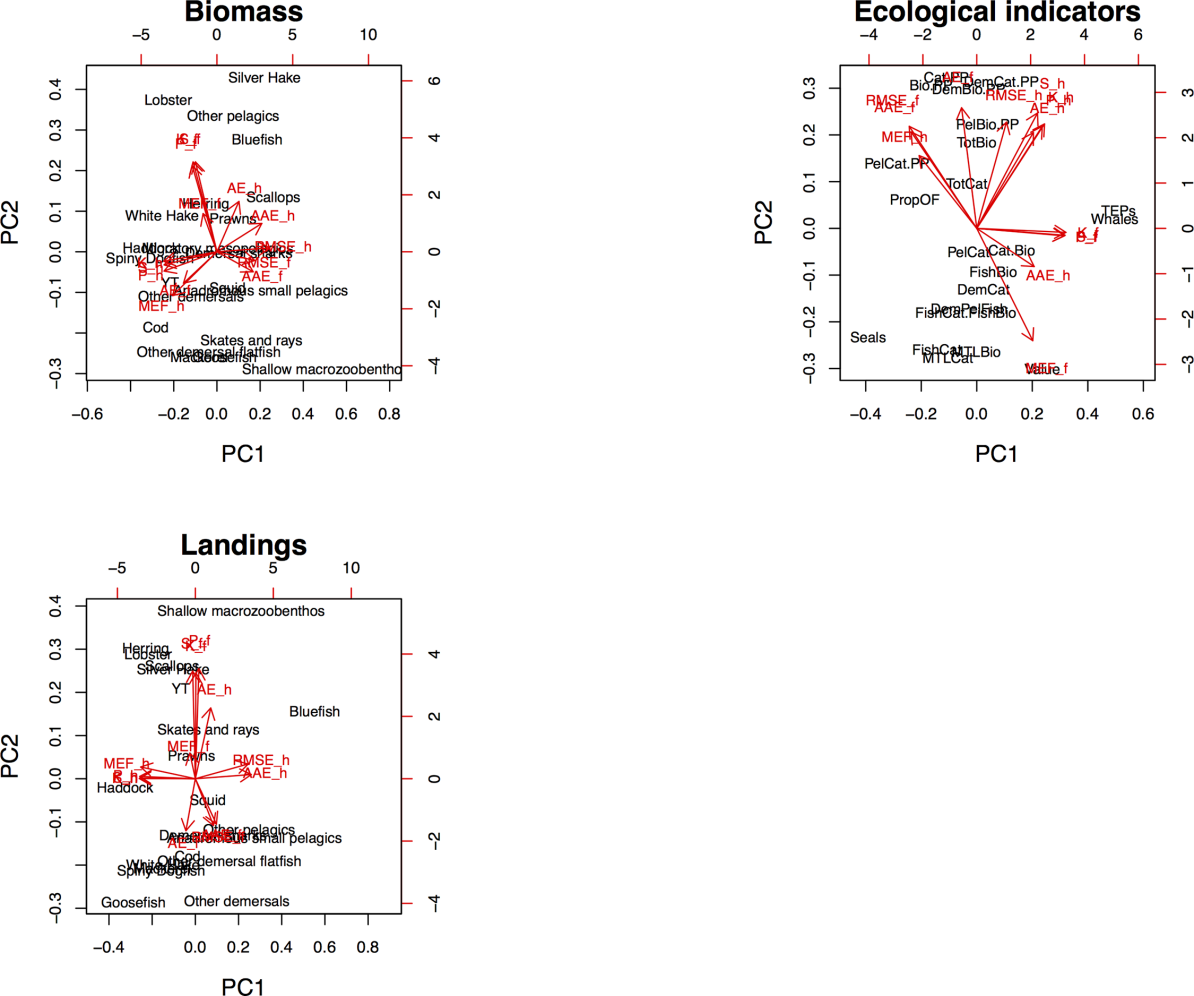
Principal component (PCA) biplot of loadings and score of the 1^st^ and 2^nd^ principal component from a PCA analysis of the different skill metrics: MEF, AE, AAE, RMSE, Pearson Correlation (P), Kendall Rank Correlation (K) and Spearman Rank Correlation(S), calculated for the hindcast (_h) and forecast (_f) for the time-series for biomass, landings and ecological indicators. Loadings for the skill metrics are shown in red while scores for the species, catches and indicators are in black.

Model performance varied across biomass, landings, and ecological indicators. Model skill across multiple metrics for biomass outputs suggests that the model performed better in hindcast for ecologically, economically, and scientifically important species like cod, haddock, and spiny dogfish (Fig 6) that were emphasized during the model calibration phase (see *Materials and Methods*). Overall, skill was higher for cod, spiny dogfish, haddock, and lobster biomass, and lower for yellowtail flounder, shallow macrozoobenthos, squid, and ‘other demersal flatfish’ biomass (the last three of these are modeled as aggregate groups rather than individual species). In general, MEF values for biomass were below 0 (poor performance) for many of the taxa groups. Hindcast biomass MEF values above 0 (indicating above average performance) were obtained for haddock, ‘migratory mesopelagics’, herring, white hake, and spiny dogfish, while in the forecast lobster, ‘other demersals’, cod, ‘other demersal flatfish’, white hake, and spiny dogfish had positive MEFs. The NEUS model had the poorest skill for the biomass of shallow macrozoobenthos, a data-poor group, as measured by all metrics (large negative MEF, high AE and AAE, and negative correlation coefficients, Fig 6). Both hindcast and forecast MEFs correlated with the Spearman, Pearson and Kendall correlation coefficients, (see PCA analysis in 1^st^ panel in) The AAE and RMSE were correlated for both the hindcast and forecast, and these two metrics were weakly correlated with AE in the hindcast, but were uncorrelated in the forecast (first panel of Fig 7). Herring, ‘other demersal flatfish’, spiny dogfish, white hake, and haddock had the five lowest hindcast AAEs, while ‘other pelagics’, ‘other demersal flatfish’, white hake, herring, silver hake, and spiny dogfish had the five lowest forecast AAEs. The same species ranked among the lowest RMSEs. AE values were more variable, making it difficult to differentiate among the lowest-values AE. Correlations >0.5 were found for the hindcast for lobster, ‘anadromous small pelagics’, haddock, ‘migratory mesopelagics’, herring, ‘other demersal flatfish’, white hake, and spiny dogfish. In the forecast, lobster, silver hake, goosefish, and yellowtail flounder showed similarly high correlations. The relationships between the five indicators were similar to those observed for the biomass data (see PCA analysis, panel 2, Fig 7).

**Fig 8:**
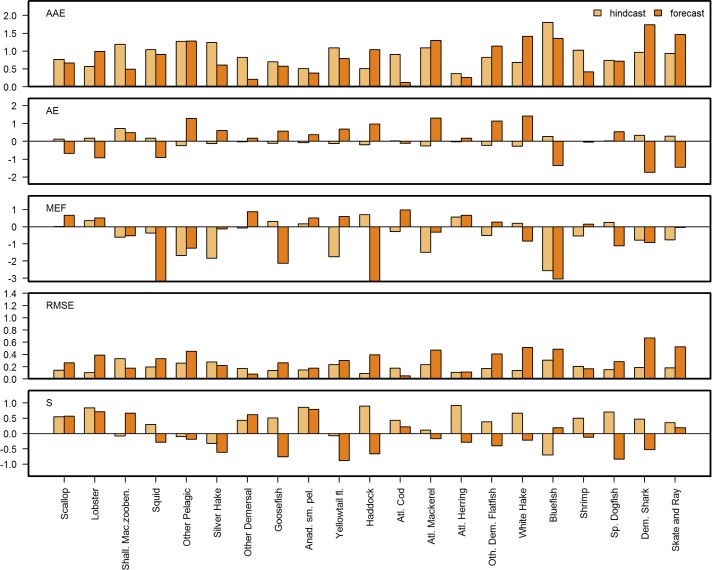
Forecast skill metrics for fisheries landings data. Pairwise comparison of forecast (_f) and hindcast (_h) skill metric performance for 5 skill metrics: MEF, AE, AAE, RMSE and S(pearman rank) Correlation for 21 species of commercial fish and shellfish in the NEUS Atlantis ecosystem model. Y-axis limited to show values between -2 and 2.

Model skill for biomass outputs did not necessarily correlate with model skill for landings, demonstrating that looking at both categories is important and confirming that the latter is not typically a suitable proxy for the former. Commercial fish species, especially pelagics, showed the best performance for model-predicted landings compared with observations (Fig 8). Above 0 threshold hindcast catch MEF values were achieved for haddock, herring, white hake, and spiny dogfish. Forecast MEFs >0 were obtained for scallop, lobsters, ‘other demersal’, ‘anadromous small pelagics’, yellowtail flounder, cod, herring, ‘other demersal flatfish’, and shrimp. Model skill for scallop and shallow macrozoobenthos landings were better than obtained for the biomass of those groups. Landings for haddock and herring showed poorer skill across metrics than for biomass.

**Fig 9:**
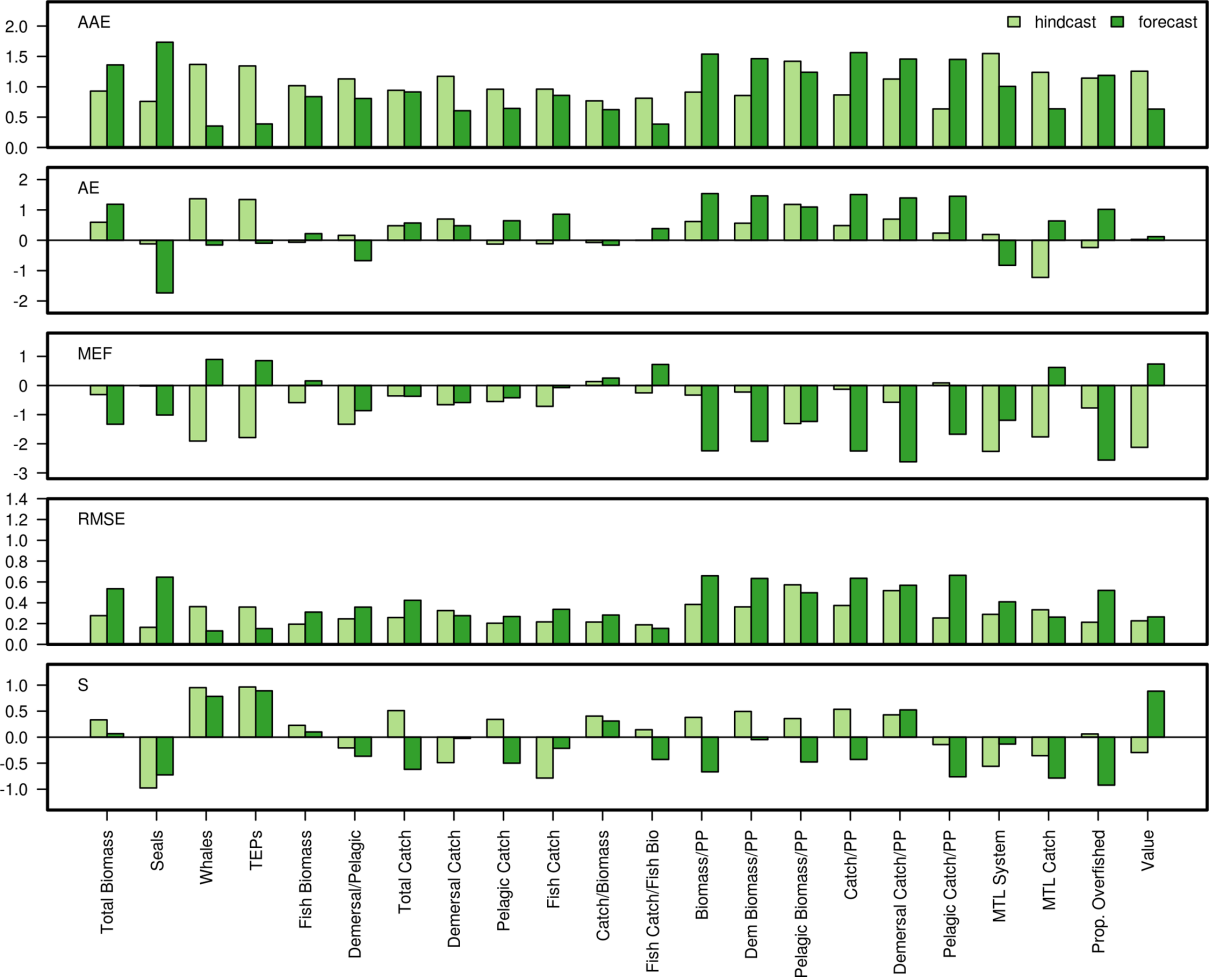
Forecast skill metrics for ecosystem indicators emulating emergent ecosystem properties. Pairwise comparison of forecast (_f) and hindcast (_h) skill metric performance for 5 skill metrics: MEF, AE, AAE, RMSE and S.(pearman rank) Correlation for 16 ecological indicators based on biomass and landings data. Y-axis limited to show values between -2 and 2.

Ecosystem indicator skill was overall lower than that for biomass and landings (Fig 9), with only ‘Catch/Biomass’ having a MEF >0 in hindcast mode. However in forecast mode seven indicators had MEFs >0 (Whales, Threatened, endangered, and protected species (TEPs), ‘Catch/Biomass’, ‘Fish Catch/Fish Biom.’, ‘MTL Catch’, and Value). Only two indicators (Whales and TEPs) had Spearman correlations >0.5 during the hindcast, increasing to three (‘Proportion overfished’) for the forecast.

The model was not originally calibrated to any ecosystem indicators so the better than average forecast MEF metrics are encouraging. Still, skill was poor for many of the ecosystem indicators such as the ratio of demersals to pelagics (‘Demersal/Pelagic Ratio’, Fig 9), likely reflecting the poor performance at the species level for some demersal groups (e.g. ‘other demersal flatfish’). Skill metrics for the mean trophic level (MTL), seal biomass, and total biomass indicators did not perform as well as fishery-oriented indicators that included landings information. However, the whale biomass indicator and the index of TEPs (which is dominated by whale biomass) performed moderately well. For the only economic indicator, value of the catch, AAE and MEF showed better performance for the forecast than the hindcast, while the other metrics showed worse performance for the forecast, especially the correlation which was very negative. The negative correlations were due to the strongly diverging time series for these indicators in the forecast period, with one time series showing increase while the other showed a decrease (see Fig 5). The PCA analysis showed that the relationship between the seven skill metrics was different for the ecosystem indicators than for the biomass and landings (bottom panel, Fig 7), with both hindcast and forecast MEFs being uncorrelated with the correlation coefficients, and RMSE and AAE showing a lower degree of correlation with each other. The principal component scores (Fig 7) show how the species, landings and ecosystem indicators with high skill scores were grouped on the 1^st^ and 2^nd^ principal component axes. Similarly, low skill components (e.g. shallow macrozoobethos) are outliers. Interpretation of the results is further complicated by species-specific catchability terms associated with scientific surveys that may introduce bias to individual species biomasses, landings, and ecosystem indicators. Further development of skill assessment methods for the NEUS model should take these caveats into account.

### Forecast vs. hindcast skill

**Fig 10:**
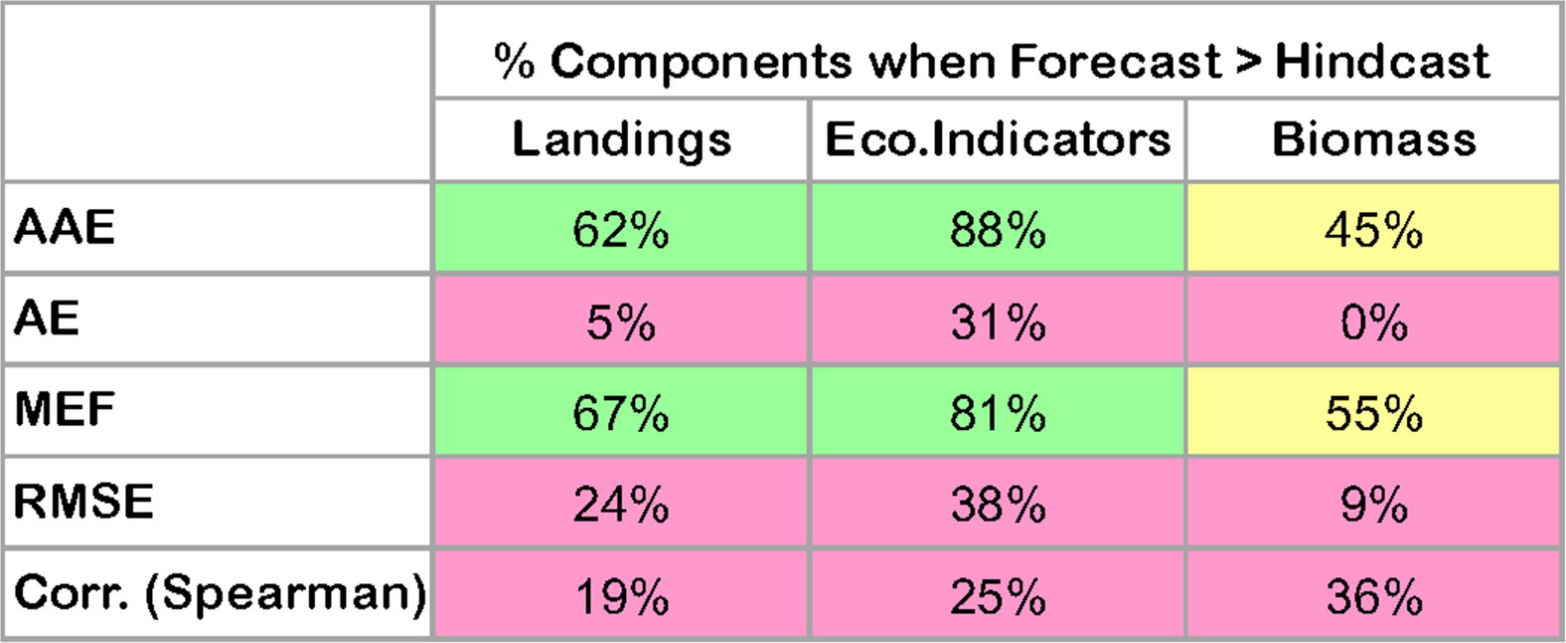
Comparison of model skill performance for hindcast (1974–2004) and the model forecast (2004–2013) for species biomass or landings and ecosystem indicators. Pink: <40%, Yellow: 40–60%, Green: >60%

Forecast skill was overall similar to hindcast skill, as shown by the metric plots (Figs 6, 8 and 9) and synthesized in Fig 10 with 39% of the forecast skill metrics falling within the same (40%-60% range of the hindcast) or better quantiles (>60%) than the hindcast. Forecasts performed better than hindcasts for the ecosystem indicators where 57% of the forecasts showed better metric performance than during the hindcast (Fig 10), with AAE and MEF indicating the most improved skill. These results are encouraging as they indicate that the model forecast performance does not degenerate, but remains stable during medium-term prediction of future conditions.

### Relationships between skill assessment metrics

Principal component analysis (Fig 7) of the skill metrics for each of the hindcast and forecasts substantiates our conceptual model (Fig 1) that the metrics measure different aspects of model skill and that several skill metrics are needed to properly analyze the performance of ecosystem models. The different correlation metrics were redundant across all three data categories. RMSE and AAE were redundant for the landings and biomass data as their loading vectors (Fig 7) point in the same direction, indicating correlation between the two. This redundancy was not observed for these metrics for the ecosystem indicators. According to our conceptual model simulations, calculated values for RMSE and AAE diverge the most when there is a trend mismatch or inverse correlation between model output and data, and are most similar when model output and observations are uncorrelated (Fig 1). The redundancy of RMSE and AAE for biomass and landings suggests that mismatches between model outputs and data were due to lack of correlations between model and output data regardless of scale. Differences between RMSE and AAE for ecosystem indicators may suggest more mismatched trends overall.

## Discussion

Evaluating the performance of mathematical models to be used in decision-making is of utmost importance; ideally we must have confidence that model predictions are reliable or robust before we can use them as a basis for management. However, assessing the skill of highly complicated models with many interacting components at large temporal and spatial scales can be daunting. Our results show that skill assessment is feasible for an end-to-end ecosystem model, and that multiple metrics are necessary to evaluate model skill. In addition, our results demonstrate that skill assessment can give end users appropriate guidance on how model outputs are best used, particularly highlighting which model results are reliable and which should be treated with caution. Finally, we recommend skill assessment as an integrated part of all model development as model skill levels can be set as *a priori* criteria before a model is accepted for use in advice or management. We discuss each of these points in more detail below.

### Skill assessment is possible for end-to-end ecosystem models

Generality, process realism, and precision are all desirable aspects of mathematical models of systems [41,42] leading to improved understanding, prediction, and modification of those systems depending upon their use. Models are generally not capable of achieving all three, and so different applications de-emphasize one quality to optimize the other two [41]. One difficulty with using end-to-end ecosystem models is that they are designed to provide overall context—generality and realism—but management systems for living marine resources also strongly desire precision [16,42]. The focus of skill assessment for end-to-end models needs to remain on generality and realism, because these are the contextual characteristics necessary for a model to be used for strategic evaluation of management options, rather than tactical management of individual species, stocks, or habitats. We therefore emphasized whether model forecast skill was expected to be at least equal to hindcast skill over multiple data categories when using multiple metrics. We also emphasized model performance for emergent ecosystem properties.

Multiple data sources allowed us to better evaluate skill in this end-to-end ecosystem model. Basing our skill assessment on two separate time-series (biomass and landings) from which we computed the emergent third (ecosystem indicators) allowed us to evaluate the NEUS model skill from three perspectives important to natural resource managers. Together these three time-series paint a 3D picture covering the biological, ecological and human-use dimensions of the model skill performance, a level that is necessary to adequately understand the complexities of a socio-ecological system. First, we were able to address model performance from a biological perspective: how good is the model at forecasting the status of single components (e.g. species) of the system? Second, understanding how well the model performs with respect to human uses of the system is important for managers, and is highlighted using fisheries landings. Finally, emergent properties of the system are important to track to characterize ecosystem structure and function, and system response to multiple pressures. These are assessed using ecosystem indicators that measure different aspects of interactions between model components. Not addressed by our assessment are discussions about which data are most important or preferred when addressing model skill, in particular the uncertainty of the observed biomass time series illustrated by the confidence bands around the survey biomass time-series in Fig 3. Appropriate weighting of data sources when assessing model performance is a key concern, and although methods exist for weighting data during model tuning that are also relevant for model skill evaluation, such weighting is often subjective [5,7].

Assessing the ecological skill of ecosystem models—how well they reproduce emergent system properties—may be more important than evaluating the single component skill, because simpler models can be applied if population dynamics for only one or a few species are of interest. Our analysis identifies the use of ecosystem indicators as one pathway to assessing model skill for emergent system properties. The benefits of using ecosystem indicators are that they are understandable even outside of specialist communities, and have been analyzed extensively in the literature [20,21,43]. There are other indicators that could also be considered [20,21]. Indicators used in the present study range from broad systemic indicators (total biomass, total catch, demersal catch, fish biomass, fish catch and pelagic catch), the narrower (seal and whale biomass) that represent the state of a range of top-predator species, to ratios (demersal / pelagic, fish catch / fish biomass, proportion overfished, ratio to primary production) and external factor metrics (value, mean trophic levels) that are calculated based on system state and external factors (e.g. definition of trophic level for each species). The broad indicators seemed to have better metric performance both in the hindcast and forecast mode, but there was large variability between the indicator and metric combinations. This variability may be reduced by improved overall model performance, but it indicates a need for using multiple indicators to monitor an ecosystem; no single indicator is superior and covers all system properties.

Long-term monitoring programs, collecting data across multiple ecosystem components and processes, are required to assess skill in an ecosystem model where dynamics take place over large spatial scales and long timescales. In our application, the end-to-end model was constructed for a data-rich system, and sufficient time had passed since model completion to use 10 years of data for skill assessment. We recognize that these circumstances may rarely be encountered, but other methods exist to examine model skill. For example, during model development, a portion of time series data may be left aside to assess skill relative to a more limited hindcast dataset [13,44]. While these approaches should not substitute for periodic assessment of model forecast skill with new observations collected after model construction, they are likely to improve both model performance and user knowledge on which model outputs should be regarded as most accurate and precise, and which might be highly uncertain.

Our skill assessment focused on properties of well-sampled ecological components, largely those of interest to humans via food production or biodiversity objectives. There are other aspects of model skill that could be assessed for an end-to-end ecosystem model, depending on how it is to be used. If data are available, both hindcast and forecast skill assessment could be completed for physical oceanographic properties, nutrients and chemical properties, primary producers, zooplankton, and benthos. Similarly, model skill in the response to management interventions (e.g. changes in fishing policy, area closures, etc.) could be assessed. Biological parameters like age and size structure of species could also be the subject of future skill assessments. Here we have compared the most readily available and longest time series data for the NEUS region, focusing on fish and upper trophic levels as a baseline skill assessment, but further work can be done especially if combined with targeted monitoring programs.

### Multiple metrics are necessary to assess model skill

Model skill is a combination of accuracy and precision and various metrics measure these in different ways. Metrics are sorely needed to discern poor fits that are not apparent to visual inspection, like perfect inverse variation and uncorrelated off-scale data (Fig 1, columns 3 and 4) that seem both similar and where a visual inspection could easily interpret the time-series plots as the model fitting the data reasonably well. Correlation metrics focus only on precision, as the model can show high correlation for results that have very low accuracy (far from the true value, see Fig 1, second column). AAE, AE, and RMSE are measures of accuracy, measuring the difference between the modeled results and the “true” (in our case observed) system state. Modeling efficiency (MEF) is the only metric we used that evaluated both precision and accuracy. No single metric fully expresses all aspects of the differences between model outputs and observations, which is both shown by the multivariate analysis NEUS model metrics (Fig 7) and the conceptual model (Fig 1). Some metrics like Spearman, Pearson, or Kendall correlation coefficients are clearly redundant, and only one of these should be used. We recommend the Spearman rank correlation coefficient, as this is non-parametric and is more commonly used than Kendall rank correlation. Spearman rank correlation has previously been used in indicator skill evaluation [21]. Our results showed that RMSE and AAE were redundant metrics for biomass and landings, but not for emergent ecosystem indicators, suggesting that both metrics should be used for a full evaluation of model performance. This supports the results from the conceptual model (Fig 1) that also indicated possible redundancy between RMSE and AAE. The AE results demonstrated that this metric is redundant with AAE and often RMSE, and is not as informative a skill metric as others. MEF is the most stringent skill assessment metric applied here, asking not just whether the scale and correlation match between model and observations, but whether the modeled time series represented an improvement over taking the mean of the observations. Conceptually, MEF is most sensitive to scale-off effects and inverse variations, and less sensitive to lack of correlation or trend mismatches (see Fig 1, bottom row), and should therefore not be used alone without the complement of a correlation metric. It is our recommendation that future end-to-end ecosystem model skill assessment use at least the four most informative skill metrics: MEF, RMSE, AAE, and Spearman rank correlation.

### Skill assessment of the Atlantis NEUS model, and recommendations for its further development

The low model skill for the majority of the components and indicators evaluated is discouraging, but this is compensated by the higher skill for the key species, and the skill in forecast mode., Our overall assessment of the results is that our results underscore those of Link et al. (2010) [19] that the Atlantis NEUS ecosystem model can realistically forecast system properties and component behaviors over a 10-year period, at least relative to our ability to reconstruct historical dynamics–which the hindcast was calibrated to. Even though MEF was less than zero for many model components and indicators, achieving MEFs higher than zero for model components is a sign that the model performs better than the long-term average, something that would not happen by chance, and is further highlighted by how model projections for key ecosystem components (e.g. spiny dogfish, haddock, cod, and white hake) fit within observed data time-series confidence bands (Fig 3). The forecast skill across data sets and metrics was comparable with the hindcast. That model forecasts were at least as skillful as hindcasts, combined with the apparent skill at predicting emergent ecosystem properties, demonstrates that this model can be useful to managers for strategic management purposes (e.g. for Management Strategy Evaluation). For some key ecosystem components and ecosystem indicators (e.g. Catch/Biomass ratio) the model performed very well for the whole time-series. Similarly, despite changes in fishery management over the past 10 years that were not included in the model, landings hindcast and forecast skill were comparable. However, the low skill for many components underscores that one cannot rely on the Atlantis NEUS model alone, and so multiple models and tools should be used to overcome the faults and limitations of each one. Managers must also carefully consider in what instances their needs are best served by a complex ecosystem model, or when simpler multispecies models would suffice. The poor model skill for many model components, catches, and indicators shows that the applicability of the Atlantis NEUS model must be evaluated in relation to each specific management issue.

The inherent complexity of ecosystem models like Atlantis prohibits statistical fitting of each individual model-predicted time-series to observed data. Link et al. [19] used qualitative visual fits when calibrating the NEUS model such that the “…*goal of this tuning was to parameterize a base scenario which reasonably captured the magnitude and overall shape of the observed biomass*, *catch and effort time series…”*, first tuning the biophysics (e.g. energy flows), then the biological components (species biomass) and finally the fisheries catches. Hindcast skill metrics showed that the model components that were the focus during the model calibration phase exhibited higher skill than the other components (see MEF scores in Figs 6 and 8 for silver hake, yellowtail flounder, cod, haddock, herring, spiny dogfish, and white hake), and were more closely grouped in the principal component biplot in Fig 7. This suggests that focused calibration across multiple species yielded acceptable skill performance, and in turn relatively robust ecosystem dynamics. With dedicated effort, ecosystem models can achieve performance that adds analytic value. Groups that performed poorly were those for which there were fewer data (e.g. small macrozoobenthos and shrimp), have biological dynamics that may not be able to be modeled by Atlantis NEUS as currently parameterized (e.g. lobster and bluefish), or may have been subject to management actions not present in the model (e.g. sea scallops which are protected by closures on Georges Bank). In some cases the model provided insight related to incorrect assumptions about observed survey data or stock assessment outputs. An example of this was for shrimp where the Atlantis ecosystem model dynamics suggested that abundance should be higher than predictions from stock assessments. Subsequent stock assessments also indicated higher levels of shrimp. Other species or groups might require modifications to the Atlantis equations to be modeled correctly, or might require modeling of implemented management actions to be included in any calibrated scenario.

### Incorporating skill metrics in model calibration

The results of the skill assessment also highlight the model parameters that should be focused on when models are initially designed, updated, and improved. For example, further calibration can be targeted to achieve MEF values greater than 0 for more model components, a much stricter criterion than those used during the original model calibration. Such strict criteria can be used both during the calibration phase of model development as well as during external evaluation and review (vetting) of models for use by management. While end-to-end ecosystem models are not intended for tactical advice on single species management, knowing the relative model skill for groups of species is useful when developing and interpreting analyses for applied management. For example, evaluating management strategies related to groundfish fishery management may be more effective in this model than evaluating strategies related to inshore benthic invertebrate fisheries or aquaculture.

### Improving and expanding ecosystem model skill assessments

Our analysis evaluated the skill of the whole model for all model areas combined, but it would have been possible–albeit analytically more demanding–to do a skill assessment at the finer spatial scale of each Atlantis model region (see Fig 2). In a practical fisheries management application, ensuring that the model has spatial skill in predicting biomass patterns is important. A finer spatial scale may also be useful to identify what causes low skill for certain model components. It would also be informative to carry out observation model simulation experiments [40] to pinpoint what data collection is most necessary to improve model skill. For the sake of parsimony we chose to keep the present analysis at a holistic whole-of-system level. A holistic level is applicable for ecosystem-level management advice and to illustrate overall model performance and applicability of the skill assessment methods. Future ecosystem model skill assessment should consider calculating skill metrics at smaller spatial scales, as well as developing methods to assess the model skill of matching time-series patterns, trends, and timing of events.

Skill assessment of end-to-end ecosystem models highlights the need for continued ecosystem monitoring across multiple scales, processes, and trophic levels. Ecosystem indicators can be calculated from model predictions for components spanning phytoplankton to whales. Unfortunately, data for many of these components are lacking for parts or all of the time-series, especially for lower trophic levels and for top predators (see Fig 5). In addition, data from scientific surveys have associated observation error and may be biased approximations of the true state of the ecosystem. These data limitations illustrate that to assess individual components, emergent system properties, model performance, and ultimately ecosystem structure and function within a region, data for a wide range of taxa are needed. To evaluate key ecosystem linkages and move towards ecosystem-based management, one can no longer be satisfied with data on only the commercially important components [12,14,15,45]. There continues to be an acute need for ecosystem surveys [28,29,46] to support ecosystem-based management.

## Conclusions

Can we do effective skill assessment on ecosystem models? Yes we can! And similarly, can we generally rely on ecosystem-models to guide us in strategic decision-making on large-scale socio-ecological questions such as climate change, pollution, etc.? Yes we can! Our results demonstrate that model performance can be relied on to give realistic representations of future system conditions (at least for key model components in a medium-term prediction such as this). Assessing the skill of complicated ecological models is both possible and practical using multiple metrics in relation to both single model components (e.g. species), human uses (e.g. fisheries landings), and emergent system properties and interconnections (ecosystem indicators). Through these we gain a 3D and multi-faceted understanding of model performance. In the hindcast the Atlantis NEUS model showed acceptable performance for key species, while for others the model had poor skill-metric performance, reflecting either poor model fit or that the observed survey data were an incorrect representation of the true system state. The forecast skill of the Atlantis NEUS model was comparable to the calibrated 40 year hindcast period, even when assessed using the Modeling Efficiency metric—the most stringent of the metrics we evaluated. This illustrates that a complicated ecosystem model like Atlantis can achieve performance that is realistic enough for use in Management Strategy Evaluation[16,47,48], although our results also point to specific needs for further refinement of the Atlantis NEUS model that will make it more useful and reliable as a tool for Management Strategy Evaluation in the Northeast US. It is difficult to directly compare our results with other complex ecosystem models, e.g. the Madingley ecosystem model [49] due to the lack of quantitative skill assessment of the latter, but compared to earth system models, the Atlantis NEUS model shows comparable AE and RMSE skill levels but lower correlation [10]. The methods we have explored and developed are relevant to a broad community of model users, in fields including economics [50,51], climate change [52–54], and ecological risk analysis [55,56]. The metrics employed can be calculated for any type of model, and assessing the skill in relation to several model data-sets and time-series is also generally applicable. Our analysis used a marine ecosystem model to develop methods and techniques that demonstrate the realism of our approach, but any complicated model fit to time-series data could have been used.

Ecosystem understanding is increasingly important in ecosystem-based management across issues such as climate change, biodiversity protection, and when managing the use of living resources. Ecosystem models have a clear role to play here, but need to be vetted for their use in management. Multi-faceted skill assessment should therefore be an integral part of developing an ecosystem model, so that models like Atlantis can play their intended role in advancing Integrated Ecosystem Assessments [57,58] and ecosystem-based management [59,60]. We assert that skill assessment can be done, it should be done, and that doing so provides benefits and credibility, illustrating the suitability and robustness of integrated ecosystem-based approaches for managing complex systems.

## Supporting Information

S1 File Decadal model skill metric analysis. (DOCX)
