# Supplementary material for: Ecosystem Model Skill Assessment. Yes We Can!
Source: PLoS One. 2016 Jan 5;11(1):e0146467. doi: 10.1371/journal.pone.0146467 (PMC4701724; doi:10.1371/journal.pone.0146467)
Supplement: S1 File — (DOCX) [file pone.0146467.s001.docx]

Supplementary Online Material to

**Ecosystem model skill assessment.**

**Yes we can!**

**Authors**: Olsen, E. , Fay, G., Gaichas, S., Gamble, R., Lucey, S., and Link, J.S.

# Decadal Model skill metric analysis

Skill metrics were calculated on a decadal basis and compared to the full hindcast (1964-2004) and to the forecast (2005-2013). Visual evaluation of Figs 1-15 revealed no temporal trend in the metric performance, especially for the first 10 years of the model run (the “no burnin” period) compared to other decades or the full hindcast.

## Biomass

Fig A Average Absolute Error (AAE) skill for the NEUS Atlantis ecosystem model biomass components for each decade from model start year (1964) to 2013 compared with the full model hindcast (black: 1964-2003). The model was tuned to observed and assessment data for the 1964-2004 (hindcast) period, while for the 2005-2013 was used in predictive mode.

Fig B Average Error (AE) skill for the NEUS Atlantis ecosystem model biomass components for each decade from model start year (1964) to 2013 compared with the full model hindcast (black: 1964-2003). The model was tuned to observed and assessment data for the 1964-2004 (hindcast) period, while for the 2005-2013 was used in predictive mode.

Fig C Modelling efficiency (MEF) skill for the NEUS Atlantis ecosystem model biomass components for each decade from model start year (1964) to 2013 compared with the full model hindcast (black: 1964-2003). The model was tuned to observed and assessment data for the 1964-2004 (hindcast) period, while for the 2005-2013 was used in predictive mode.

Fig D Root Mean Squared Error (RMSE) skill for the NEUS Atlantis ecosystem model biomass components for each decade from model start year (1964) to 2013 compared with the full model hindcast (black: 1964-2003). The model was tuned to observed and assessment data for the 1964-2004 (hindcast) period, while for the 2005-2013 was used in predictive mode.

Fig E Spearman rank correlation (S) skill for the NEUS Atlantis ecosystem model biomass components for each decade from model start year (1964) to 2013 compared with the full model hindcast (black: 1964-2003). The model was tuned to observed and assessment data for the 1964-2004 (hindcast) period, while for the 2005-2013 was used in predictive mode.

## Landings

Fig F Average Absolute Error (AAE) skill for the NEUS Atlantis ecosystem model fishery landings for each decade from model start year (1964) to 2013 compared with the full model hindcast (black: 1964-2003). The model was tuned to observed and assessment data for the 1964-2004 (hindcast) period, while for the 2005-2013 was used in predictive mode.

Fig G Average Error (AE) skill for the NEUS Atlantis ecosystem model fishery landings for each decade from model start year (1964) to 2013 compared with the full model hindcast (black: 1964-2003). The model was tuned to observed and assessment data for the 1964-2004 (hindcast) period, while for the 2005-2013 was used in predictive mode.

Fig H Modelling Efficiency (MEF) skill for the NEUS Atlantis ecosystem model fishery landings for each decade from model start year (1964) to 2013 compared with the full model hindcast (black: 1964-2003). The model was tuned to observed and assessment data for the 1964-2004 (hindcast) period, while for the 2005-2013 was used in predictive mode.

Fig I Root Mean Squared Error (RMSE) skill for the NEUS Atlantis ecosystem model fishery landings for each decade from model start year (1964) to 2013 compared with the full model hindcast (black: 1964-2003). The model was tuned to observed and assessment data for the 1964-2004 (hindcast) period, while for the 2005-2013 was used in predictive mode.

Fig J Spearman rank correlation (S) skill for the NEUS Atlantis ecosystem model fishery landings for each decade from model start year (1964) to 2013 compared with the full model hindcast (black: 1964-2003). The model was tuned to observed and assessment data for the 1964-2004 (hindcast) period, while for the 2005-2013 was used in predictive mode.

## Ecosystem indicators

Fig K Average Absolute Error (AAE) skill for the NEUS Atlantis ecosystem indicators for each decade from model start year (1964) to 2013 compared with the full model hindcast (black: 1964-2003). The model was tuned to observed and assessment data for the 1964-2004 (hindcast) period, while for the 2005-2013 was used in predictive mode.

Fig L Average Error (AE) skill for the NEUS Atlantis ecosystem indicators for each decade from model start year (1964) to 2013 compared with the full model hindcast (black: 1964-2003). The model was tuned to observed and assessment data for the 1964-2004 (hindcast) period, while for the 2005-2013 was used in predictive mode.

Fig M Modelling Efficiency (MEF) skill for the NEUS Atlantis ecosystem indicators for each decade from model start year (1964) to 2013 compared with the full model hindcast (black: 1964-2003). The model was tuned to observed and assessment data for the 1964-2004 (hindcast) period, while for the 2005-2013 was used in predictive mode.

Fig N Root Mean Squared Error (RMSE) skill for the NEUS Atlantis ecosystem indicators for each decade from model start year (1964) to 2013 compared with the full model hindcast (black: 1964-2003). The model was tuned to observed and assessment data for the 1964-2004 (hindcast) period, while for the 2005-2013 was used in predictive mode.

Fig O Spearman rank correlation (S) skill for the NEUS Atlantis ecosystem indicators for each decade from model start year (1964) to 2013 compared with the full model hindcast (black: 1964-2003). The model was tuned to observed and assessment data for the 1964-2004 (hindcast) period, while for the 2005-2013 was used in predictive mode.
